# A hemicryptophane with a triple-stranded helical structure

**DOI:** 10.3762/bjoc.14.162

**Published:** 2018-07-24

**Authors:** Augustin Long, Olivier Perraud, Erwann Jeanneau, Christophe Aronica, Jean-Pierre Dutasta, Alexandre Martinez

**Affiliations:** 1 Aix Marseille Univ, CNRS, Centrale Marseille, iSm2, Marseille, France; 2 Laboratoire de Chimie École Normale Supérieure de Lyon, CNRS, UCBL46, Allée d'Italie, F-69364 Lyon, France; 3 LMI-UMR 5615 CNRS / UCBL, Université Claude Bernard Lyon 1, 6 rue victor Grignard, 69622 Villeurbanne cedex, France

**Keywords:** CTV, hemicryptophanes, organic cages, triple helical structure

## Abstract

A hemicryptophane cage bearing amine and amide functions in its three linkers was synthesized in five steps. The X-ray molecular structure of the cage shows a triple-stranded helical arrangement of the linkers stabilized by intramolecular hydrogen bonds between amide and amine groups. The chirality of the cyclotriveratrylene unit controls the propeller arrangement of the three aromatic rings in the opposite part of the cage. ^1^H NMR studies suggest that this structure is retained in solution.

## Introduction

Among the remarkable architectures found in biological systems, those presenting a triple helical arrangement are of particular interest. Beside its classical double strand structure formed by Watson–Crick base pairing, DNA can also organize in a triple helical fashion [1]. These three-stranded structures of DNA naturally occur and play important roles in regulating gene function and DNA metabolism. Collagen, the most abundant protein in animals, also adopts a triple helical structure: three parallel peptide chains coil about each other in a triple stranded left-handed helical structure. Its high thermal and mechanical stability results mainly from the numerous hydrogen bonds found in the triple helix framework [2]. Bioinspired structures, based on peptide backbones, have been built, allowing a better understanding of the properties of this biological system and giving rise to numerous applications ranging from artificial collagenous biomaterials to peptides for therapeutic uses [3–5].

Recently, molecular cages presenting a triple helical structure have aroused a considerable interest [6–8]: for instance, Malik et al. described the synthesis and recognition properties of an organic cage including three helicene moieties in its arms [9]. This cage presents a triple stranded helical structure with the framework fully twisted due to the arrangement of the three helicene units in a propeller fashion. Other recent examples are the triple-stranded phenylene cages presenting a helical rod-like shape synthesized by Kirsche et al. [10].

Hemicryptophanes are chiral covalent cages combining a cyclotriveratrylene (CTV, north part) unit with another *C*_3_ symmetrical moiety (south part). They display recognition properties toward neurotransmitters and carbohydrates, and can act as molecular switches and supramolecular catalysts [11]. Their *C*_3_ symmetry makes them promising candidates to build molecular cages displaying a triple helical arrangement of the linkers. Furthermore, we have previously reported that the chirality unit in the south part and the chirality of the CTV unit in the north part influence each other, suggesting that the chirality of the CTV moiety could control the helical arrangement of the linkers [12–13].

We herein report the synthesis of the hemicryptophane **1** bearing both amide and amine functions in its three likers. In solution, the ^1^H NMR spectrum shows a *C*_3_ symmetrical cage, which is also observed in the solid state by X-ray crystallography. Moreover, in the solid, amide and amine functional groups of different linkers interact through hydrogen bonding, leading to a triple helical arrangement of the linkers. The CTV unit is also found to strongly control the chirality of this triple helices since the CTV with a *P* (respectively *M*) configuration induces a Δ (respectively Λ) chirality of the propeller-like arrangement of the linkers.

## Results and Discussion

### Synthesis of cage **1**

**Scheme 1:**
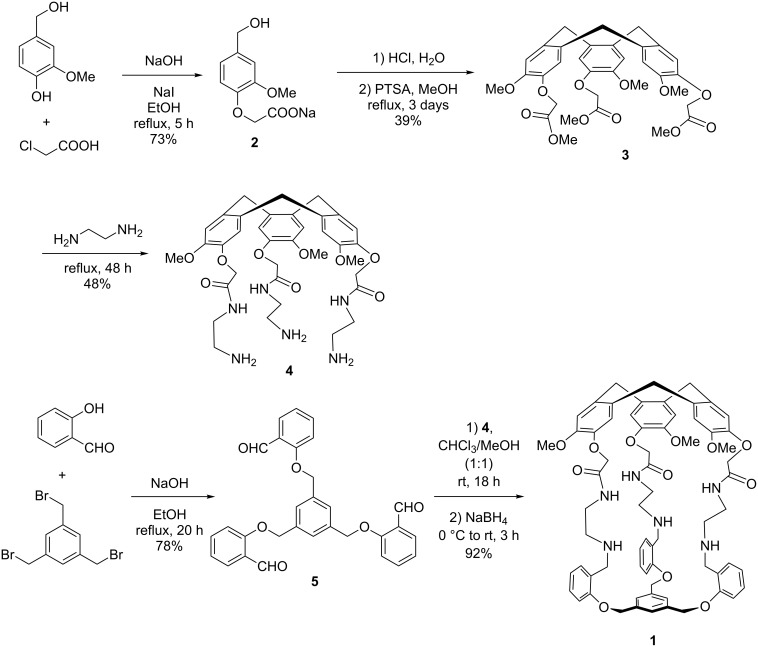
Synthesis of **1**.

According to the synthetic pathway shown in Scheme 1, hemicryptophane host **1** was obtained in five steps [14–15]. Alkylation of vanillyl alcohol by chloroacetic acid in ethanol under reflux afforded **2** in 73% yield. The CTV triester was obtained by adding first one equivalent of HCl and then a catalytic amount of *para*-toluenesulfonic acid in methanol to compound **2**. Then, compound **3** was reacted with ethylenediamine, providing **4** in 48% yield. The reaction between 1,3,5-tris(bromomethyl)benzene and 2-hydroxybenzaldehyde provided the precursor of the south unit **5** in 78% yield. Finally, a [1 + 1] macrocyclization between **4** and **5** was achieved by a reductive amination in a 1:1 CHCl_3_/MeOH mixture. A remarkable yield of 92% was obtained for this last step. As this kind of reductive amination has been proved to be under thermodynamic control, the resulting intermediate cage bearing three imine functions is highly sable [12–13]. Hydrogen bonds between the amide group and the formed imine function could account for the high stability of this intermediate, shifting the equilibrium between the different oligomers and the cage in favor of this latter (vide infra).

### ^1^H NMR of cage **1**

**Figure 1:**
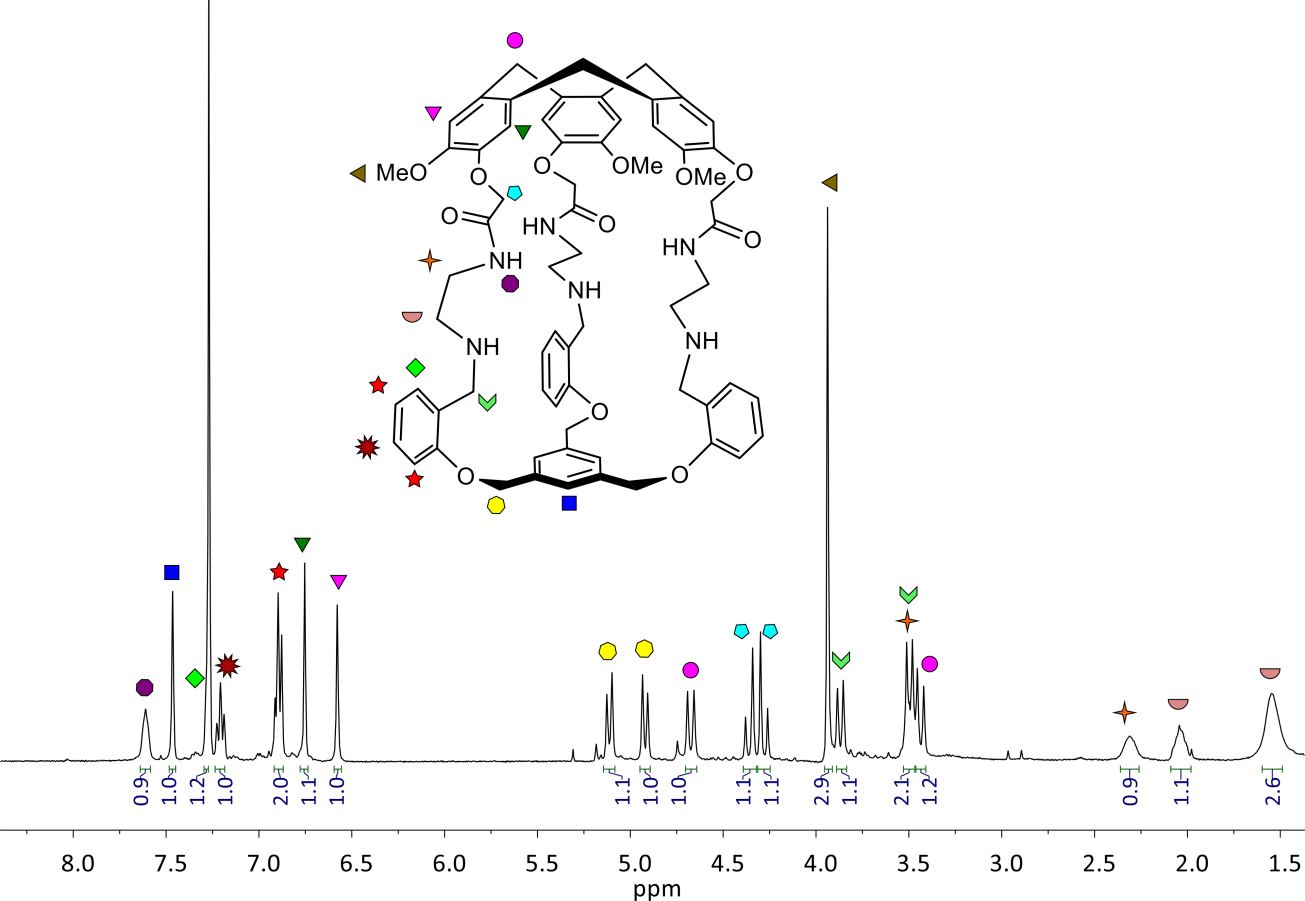
^1^H NMR spectrum of **1** (400 MHz, CDCl_3_).

The ^1^H NMR spectrum of hemicryptophane **1** in CDCl_3_ shows that this host presents, on average, a *C_3_* symmetry in solution (Figure 1). The characteristic signals of the CTV unit can be observed: one signal for the OMe group at 3.94 ppm, two singlets for the aromatic protons at 6.58 and 6.75 ppm and the expected AB systems for the CH_2_ bridges at 4.67 and 3.44 ppm. The aromatic protons of the benzene ring in the south part of the cage and the corresponding diastereotopic CH_2_ bridges displays a singlet at 7.46 ppm and two doublets at 5.11 and 4.92 ppm, respectively. The signals of the aromatic protons of the linkers give two doublets and two triplets between 6.75 and 7.2 ppm, whereas the diastereotopic aliphatic protons exhibit expected broad multiplets between 1.50 ppm and 2.36 ppm. The Ar–CH_2_–NH diastereotopic protons appear as two doublets at 3.87 and 3.49 ppm.

### Structure of cage **1**

**Figure 2:**
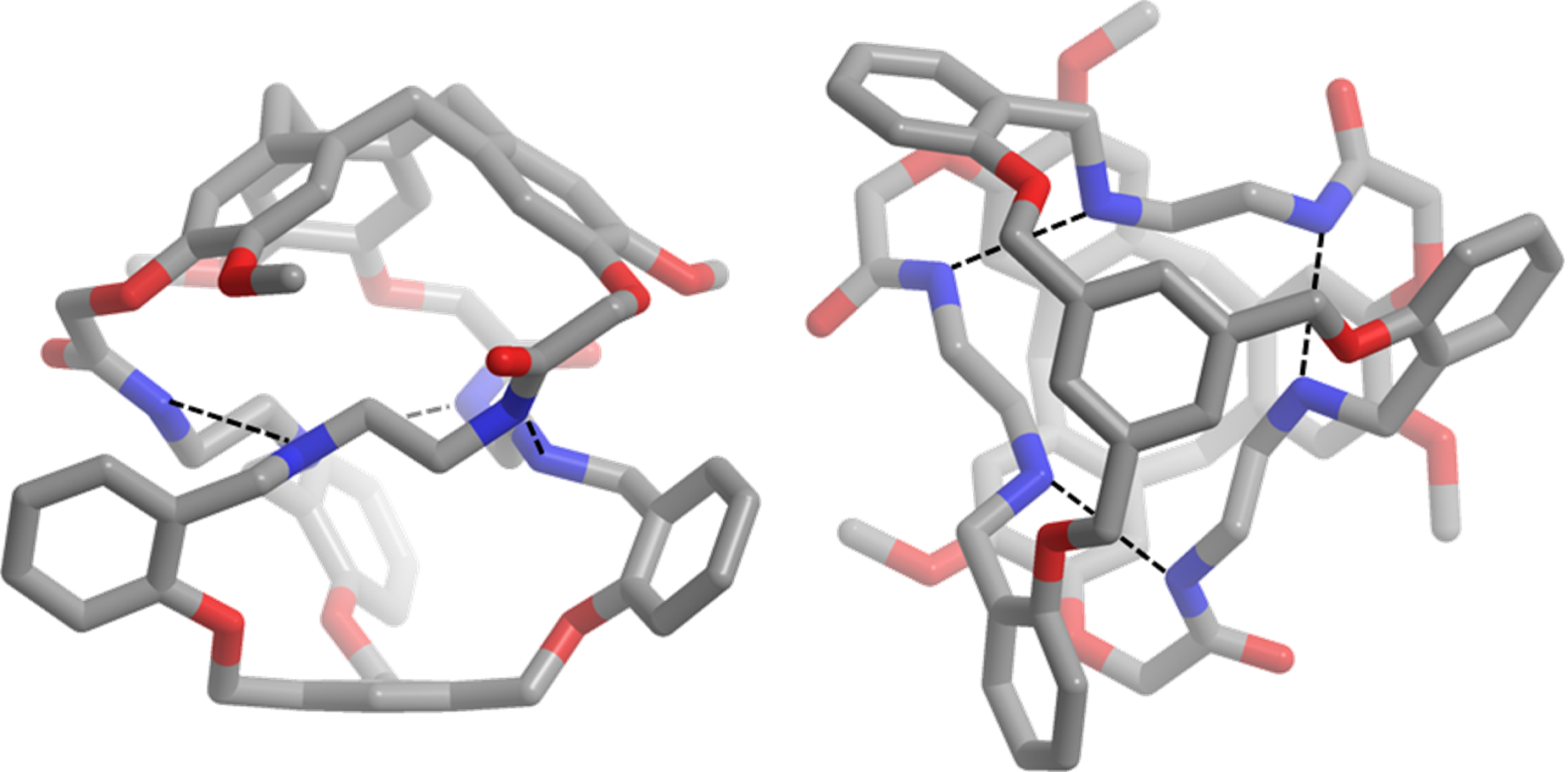
X-ray molecular structure of **1**. Hydrogen atoms are omitted for clarity; dashed lines represent hydrogen bonds.

Slow evaporation of a solution of cage **1** in a 1:1 mixture of CHCl_3_/MeOH affords crystals suitable for X-ray diffraction. In the solid, the hemicryptophane cage presents a perfect *C*_3_ symmetry (Figure 2). Further examination of the crystal structure reveals that **1** adopts a chiral conformation where the three linkers are twisted into a triple helix with the lone pair of the amines and the amide hydrogen atoms oriented toward the cavity, while the amide oxygen atoms are oriented outwards. Intramolecular hydrogen bonding between the nitrogen of the amine function of one linker with the N–H of the amide group of another arm can account for this helical structure (N_amine_···N_amide_ distances of 2.97 Å). This structure sheds light on the excellent yield achieved in the last step of the synthesis. Indeed, such intramolecular hydrogen bonding probably also occurred in the imine precursor, accounting for its high thermodynamic stability compared to oligomers that could also be formed during the reaction between **4** and **5**.

Interestingly, one can also see that the CTV with the *P* configuration (respectively *M*) imposes a Δ (respectively Λ) propeller-like arrangement of the lateral arms, with a 120° turn around the *C*_3_ axis of the molecule (Figure 2). This also underlines how the chirality of the CTV unit propagates along the linkers to induce the propeller shape of the three aromatic rings in the south part of the hemicryptophane. This remote control of the helicity of the southern part by that of the northern CTV unit, through nine bonds, is allowed by this specific triple stranded helical structure, which induces a strong twist of the whole framework.

**Figure 3:**
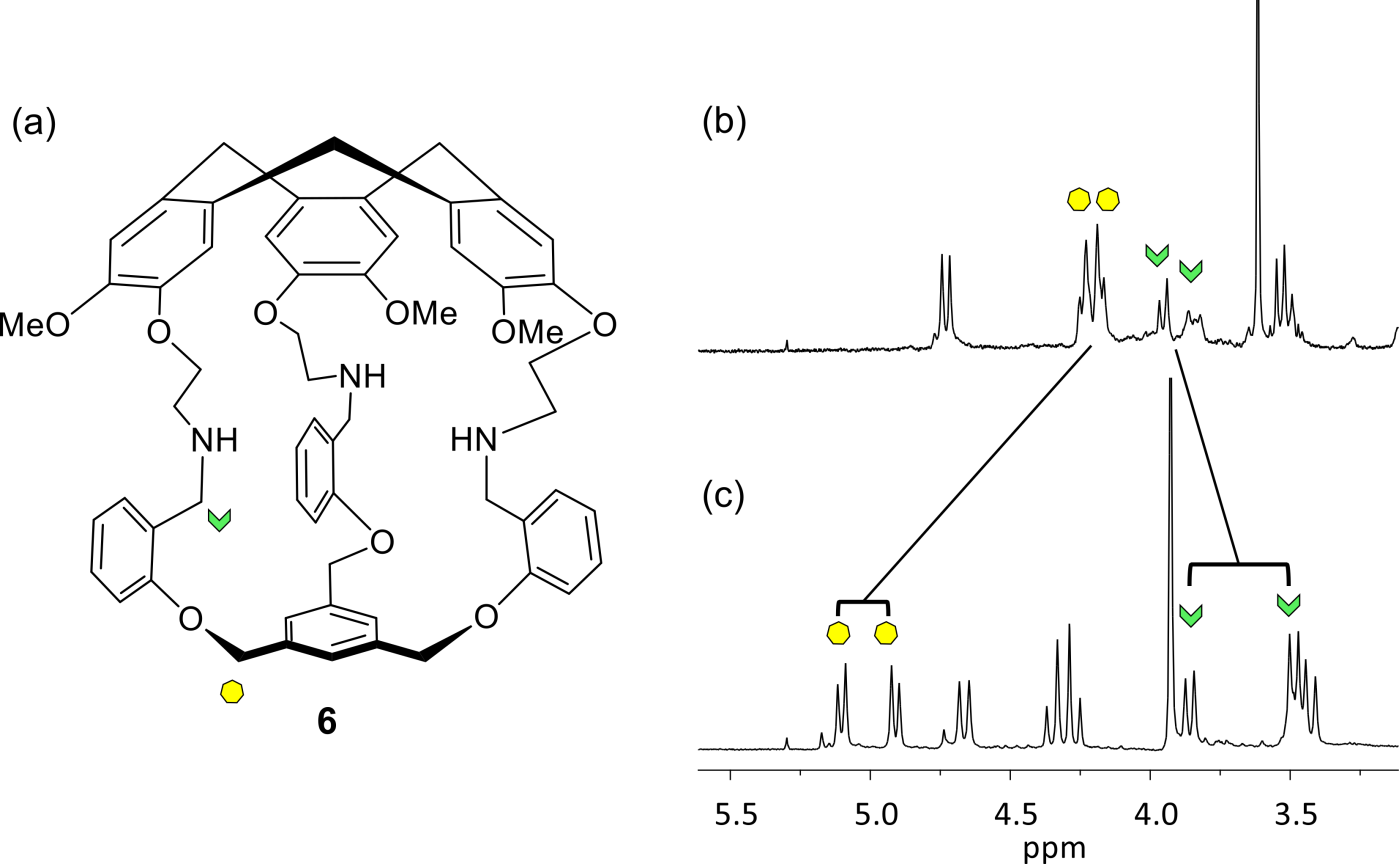
(a) Structure of compound **6**. (b) ^1^H NMR of **6** (CDCl_3_, 400 MHz). (c) ^1^H NMR of **1** (CDCl_3_, 400 MHz).

This helical arrangement probably also occurred in solution. Indeed, the south part of cage **1** is similar to that of cage **6** (Figure 3) and the comparison between the signals of the CH_2_–NH and Ar–O–CH_2_–Ar protons of cage **6** with those of cage **1** shows respectively downfield and highfield shifts of around −0.2 ppm and more than +0.8 ppm, respectively [16]. Moreover, the chemical shift differences of the two diastereotopic protons of the two AB systems are much greater for **1** than for **6** with Δδ = 0.38 ppm for the CH_2_NH and of Δδ = 0.19 ppm for the Ar–O–CH_2_–Ar of **1**, compared to 0.07 ppm and 0.05 ppm for **6**, respectively (Figure 3). This is consistent with a more rigid structure of cage **1** in solution, as suggested by the solid-state structure.

## Conclusion

In summary, we have described the synthesis of a new hemicryptophane organic cage, which adopts a triple helical structure because of the propeller-like arrangement of its three linkers. The chirality of the CTV was shown to control that of the whole helical cage structure, since only *P-*Δ and *M-*Λ enantiomers were observed in solid state. NMR studies suggest that this propeller-like arrangement also occurs in solution. Further investigations are in progress in order to propagate the chirality of the CTV to even more remote opposite sites through the formation of such triple helical structures.

## Supporting Information

File 1 Procedures for the synthesis of compounds **1**–**5**; ^1^H, ^13^C NMR, spectra mass spectra of compound **1** and crystallographic data.

## References

[1] Jain A, Wang G, Vasquez K M (2008). Biochimie.

[2] Shoulders M D, Raines R T (2009). Annu Rev Biochem.

[3] Hamley I W (2017). Chem Rev.

[4] Pekkanen A M, Mondschein R J, Williams C B, Long T E (2017). Biomacromolecules.

[5] Montero de Espinosa L, Meesorn W, Moatsou D, Weder C (2017). Chem Rev.

[6] Míguez-Lago S, Llamas-Saiz A L, Cid M M, Alonso-Gómez J L (2015). Chem – Eur J.

[7] Yamakado R, Mikami K, Takagi K, Azumaya I, Sugimoto S, Matsuoka S-i, Suzuki M, Katagiri K, Uchiyama M, Muranaka A (2013). Chem – Eur J.

[8] Ikeda A, Udzu H, Zhong Z, Shinkai S, Sakamoto S, Yamaguchi K (2001). J Am Chem Soc.

[9] Malik A U, Gan F, Shen C, Yu N, Wang R, Crassous J, Shu M, Qiu H (2018). J Am Chem Soc.

[10] Sato H, Bender J A, Roberts S T, Krische M J (2018). J Am Chem Soc.

[11] Zhang D, Martinez A, Dutasta J-P (2017). Chem Rev.

[12] Chatelet B, Joucla L, Padula D, Di Bari L, Pilet G, Robert V, Dufaud V, Dutasta J-P, Martinez A (2015). Org Lett.

[13] Gosse I, Robeyns K, Bougault C, Martinez A, Tinant B, Dutasta J-P (2016). Inorg Chem.

[14] Vériot G, Dutasta J-P, Matouzenko G, Collet A (1995). Tetrahedron.

[15] Perraud O, Robert V, Gornitzka H, Martinez A, Dutasta J-P (2012). Angew Chem, Int Ed.

[16] Long A, Perraud O, Albalat M, Robert V, Dutasta J-P, Martinez A (2018). J Org Chem.

