# Supplementary material for: A hemicryptophane with a triple-stranded helical structure
Source: Beilstein J Org Chem. 2018 Jul 24;14:1885–9. doi: 10.3762/bjoc.14.162 (PMC6071729; doi:10.3762/bjoc.14.162)
Supplement: File 1 — Procedures for the synthesis of compounds 1–5; 1H, 13C NMR, spectra mass spectra of compound 1 and crystallographic data. [file Beilstein_J_Org_Chem-14-1885-s001.pdf]

Supporting Information

for

A hemicryptophane with a triple-stranded helical structure

Augustin Long[1], Olivier Perraud[2], Erwann Jeanneau[3], Christophe Aronica[3], Jean-Pierre Dutasta[2] and Alexandre Martinez*[1]

Address: [1]Aix Marseille Univ, CNRS, Centrale Marseille, iSm2, Marseille, France; [2]Laboratoire de Chimie École Normale Supérieure de Lyon, CNRS, UCBL46, Allée d'Italie, F-69364 Lyon, France and [3]LMI-UMR 5615 CNRS / UCBL, Université Claude Bernard Lyon 1, 6 rue victor Grignard, 69622 Villeurbanne cedex, France

Email: Alexandre A. Martinez - alexandre.martinez@centrale-marseille.fr

* Corresponding author

Procedures for the synthesis of compounds 1–5; ^{1}H, ^{13}C NMR, spectra mass spectra of compound 1 and crystallographic data

1. Synthesis and materials	S2
1.1. Chemicals and materials	S2
1.2. Compound 2.[1,2]	S2
1.3. Triester CTV derivative 3.[2]	S2
1.4. Triamide-triamine CTV derivative 4.[1,2]	S2
1.5. Compound 5.[3]	S3
1.6. Hemicryptophane **1**.	S3
2. NMR and mass spectra of hemicryptophane **1**	S4
3. Crystallographic data for hemicryptophane **1**	S9
References	S14

1. Synthesis and materials

1.1. Chemicals and materials

Starting material and solvents were of commercial grade and were used without further purification. Known compounds were characterized by ^{1}H NMR and ^{13}C NMR and compared with previously described spectra. New products were characterized by ^{1}H NMR, ^{13}C NMR, 2D experiments (COSY, HSQC, HMBC), IR, melting points, HRMS. ^{1}H NMR and ^{13}C NMR spectra were recorded at 298 K on a Bruker Avance III HD 300 MHz spectrometer and a Bruker Avance III HD 400 MHz. ^{1}H NMR and ^{13}C NMR chemical shifts δ are reported in ppm referenced to the protonated residual solvent signal. TLC was carried out with Merck silica gel 60 F_{254} plates. Chromatography was performed with Merck 60 A (0.040–0.063 mm) silica gel. Melting points were determined with a Büchi Melting Point B-545. IR spectra were obtained using a Bruker Alpha Platinium ATR. HRMS (ESIMS) were performed on a QStar Elite (Applied Biosystems SCIEX) mass spectrometer with API and spectra were obtained with TOF analysis. Measurements were realized with two internal standards.

1.2. Compound 2.[1,2]

Compound **1** was synthesized according to the previously reported procedure. In a stirred solution of chloroacetic acid (22.8 g, 241 mmol) in EtOH (170 mL) and a 35% NaOH aqueous solution (25 mL) was added vanillyl alcohol (35.0 g, 227 mmol), NaI (0.38 g, 2.5 mmol) and a 35% NaOH aqueous solution (25 mL). The mixture was refluxed for 5 hours and stored at 4 °C during a night. The precipitate was filtered off on a frit and was recrystallized in water (100 mL). Compound **2** was obtained as a white solid (38.1 g, 73%). ^{1}H NMR spectrum was consistent with literature.

^{1}H NMR (D_2O, 300 MHz, 298 K) δ 7.16 (s, 1H), 7.04 (d, *J* = 8.2 Hz, 1H), 6.94 (d, *J* = 8.2 Hz, 1H), 4.66 (s, 2H), 4.58 (s, 2H), 3.98 (s, 3H).

1.3. Triester CTV derivative 3.[2]

The cyclization was performed according to a literature procedure. Concentrated HCl (10 mL) was added to a solution of compound **2** (20.9 g, 89.2 mmol) in water (50 mL). The mixture was stirred for 1 h at rt and solvent were removed under reduced pressure to yield a brown oil. MeOH (150 mL) and PTSA (250 mg, 14.5 mmol) were added and the mixture was refluxed for 3 days. The resulting precipitate was filtered off on a frit and washed with cold MeOH to yield CTV derivative **3** as a white powder (7.44 g, 39%). Characterizations of CTV derivative **3** were consistent with reported data.

^{1}H NMR ($CDCl_3$, 300 MHz, 298 K) δ 6.91 (s, 3H), 6.82 (s, 3H), 4.71 (d, *J* = 13.8 Hz, 3H), 4.61 (d, *J* = 5.0 Hz, 6H), 3.89 (s, 9H), 3.73 (s, 9H), 3.52 (d, *J* = 14 Hz, 3H); ^{13}C NMR ($CDCl_3$, 75 MHz, 298 K) 170.5, 149.2, 146.4, 134.7, 132.1, 118.2, 113.5, 67.2, 56.1, 52.0, 36.8.

1.4. Triamide-triamine CTV derivative 4.[1,2]

CTV derivative **4** was obtained according reported conditions. In a 500 mL round bottom flask, CTV derivative **3** (2.44 g, 3.91 mmol) was dissolved in ethylenediamine (150 mL). The mixture was refluxed for 48 h. The solution was cooled to rt, poured in water (500 mL) and stored at 4 °C for a night. The resulting precipitate was filtered, washed with water and dried under vacuum. Triamide-

triamine CTV derivative **4** was obtained as a white solid (1.31 g, 48%). Characterizations were consistent with literature.

^{1}H NMR ($CDCl_3$, 300 MHz, 298 K) *δ* 7.67 (br s, 3H), 6.84 (s, 3H), 6.76 (s, 3H), 4.73 (d, *J* = 13.6 Hz, 3H), 4.61 (d, *J* = 15.5 Hz, 3H), 4.48 (d, *J* = 15.5 Hz, 3H), 3.97 (s, 9H), 3.52 (d, *J* = 13.6 Hz, 3H), 3.46–3.36 (m, 3H), 3.11–2.98 (m, 3H), 2.47–2.34 (m, 3H), 2.26–2.14 (m, 3H); ^{13}C NMR ($CDCl_3$, 75 MHz, 298 K) 169.8, 147.9, 145.1, 133.8, 131.6, 114.8, 112.9, 69.1, 57.1, 42.2, 41.4, 36.7.

1.5. Compound 5.[3]

The C_3 trialdehyde moiety **5** was synthesized according reported procedure.[3] NaOH (99 mg, 2.48 mmol) was added to a solution of salicylaldehyde (237 µL, 2.28 mmol) in absolute ethanol (30 mL). After stirring during 40 minutes at rt, a solution of 1,3,5-tris(bromomethyl)benzene (227 mg, 0.635 mmol) in absolute ethanol (7 mL) was added and the mixture was refluxed for 20 h. The reaction was cooled to 4 °C overnight. The solid was filtered off and washed with cold ethanol and cold water to afford trialdehyde **5** as a white powder (248 mg, 78%).

^{1}H NMR ($CDCl_3$, 300 MHz, 298 K) *δ* 10.53 (s, 3H), 7.87 (dd, *J* = 7.7; 1.7 Hz, 3H), 7.58–7.52 (m, 6H), 7.07 (dd, *J* = 17.2; 8.1 Hz, 6H), 5.25 (s, 6H); ^{13}C NMR ($CDCl_3$, 75 MHz, 298 K) 189.7, 160.8, 137.6, 136.1, 129.0, 126.0, 125.4, 121.5, 113.1, 70.2.

1.6. Hemicryptophane 1.

In a 500 mL round bottom flask was added triamide-triamine CTV derivative **4** (316 mg, 0.446 mmol) dissolved in a 1/1 mixture of $CHCl_3$/MeOH (150 mL). Under stirring at rt, a solution of C_3 trialdehyde **5** (198 mg, 0.412 mmol) in the same mixture of solvent (80 mL) was added dropwise reaction mixture was stirred at rt for 18 h. After cooling at 0 °C, $NaBH_4$ (400 mg, 10.4 mmol) was slowly added. The mixture was stirred at rt during 3 h and solvents were evaporated under reduced pressure. The crude residue was dissolved in $CHCl_3$ (70 mL) and washed with a 10% NaOH solution (70 mL). Aqueous phase was extracted with $CHCl_3$ (2 × 40 mL) and combined organic layers were washed with a 10% NaOH solution (2 × 70 mL) and dried over $MgSO_4$. Organic solvent was removed under reduced pressure to afford a yellowish powder purified by silica gel column chromatography ($CHCl_3$/MeOH/Et_3N, gradient from 98:1:2 to 90:8:2). Evaporation of the corresponding fractions yield to hemicryptophane **1** as a white solid (432 mg, 92%).

R_f = 0.22 ($CHCl_3$/MeOH/Et_3N : 95/3/2); m.p. = 148–149 °C; ^{1}H NMR ($CDCl_3$, 400 MHz, 298 K) *δ* 7.61 (br s, 3H), 7.46 (s, 3H), 7.30–7.28 (m, 3H), 7.21 (td, *J* = 7.8; 1.3 Hz, 3H), 6.93–6.86 (m, 6H), 6.75 (s, 3H), 6.58 (s, 3H), 5.11 (d, *J* = 11.0 Hz, 3H), 4.92 (d, *J* = 11.0 Hz, 3H), 4.67 (d, *J* = 13.8 Hz, 3H), 4.36 (d, *J* = 15.3 Hz, 3H), 4.28 (d, *J* = 15.3 Hz, 3H), 3.94 (s, 9H), 3.87 (d, *J* = 12.4 Hz, 3H), 3.53–3.47 (m, 6H), 3.44 (d, *J* = 13.8 Hz, 3H), 2.36–2.26 (m, 3H), 2.09–1.99 (m, 3H), 1.56–1.50 (m, 3H); ^{13}C NMR ($CDCl_3$, 100 MHz, 298 K) 168.5, 156.9, 147.4, 145.3, 138.4, 133.0, 131.5, 130.6, 128.7, 128.5, 127.0, 121.2, 113.8, 112.9, 111.6, 70.0, 68.0, 55.9, 48.7, 47.4, 38.4, 36.5; IR σ = 3319, 2918, 2860, 1660, 1604, 1519, 1451 cm^{-1}; HRMS ESI-MS (TOF) *m/z*: observed 1141.5283 $[M+H]^+$ (calculated: 1141.5281 for $C_{66}H_{73}N_6O_{12}$).

2. NMR and mass spectra of hemicryptophane 1

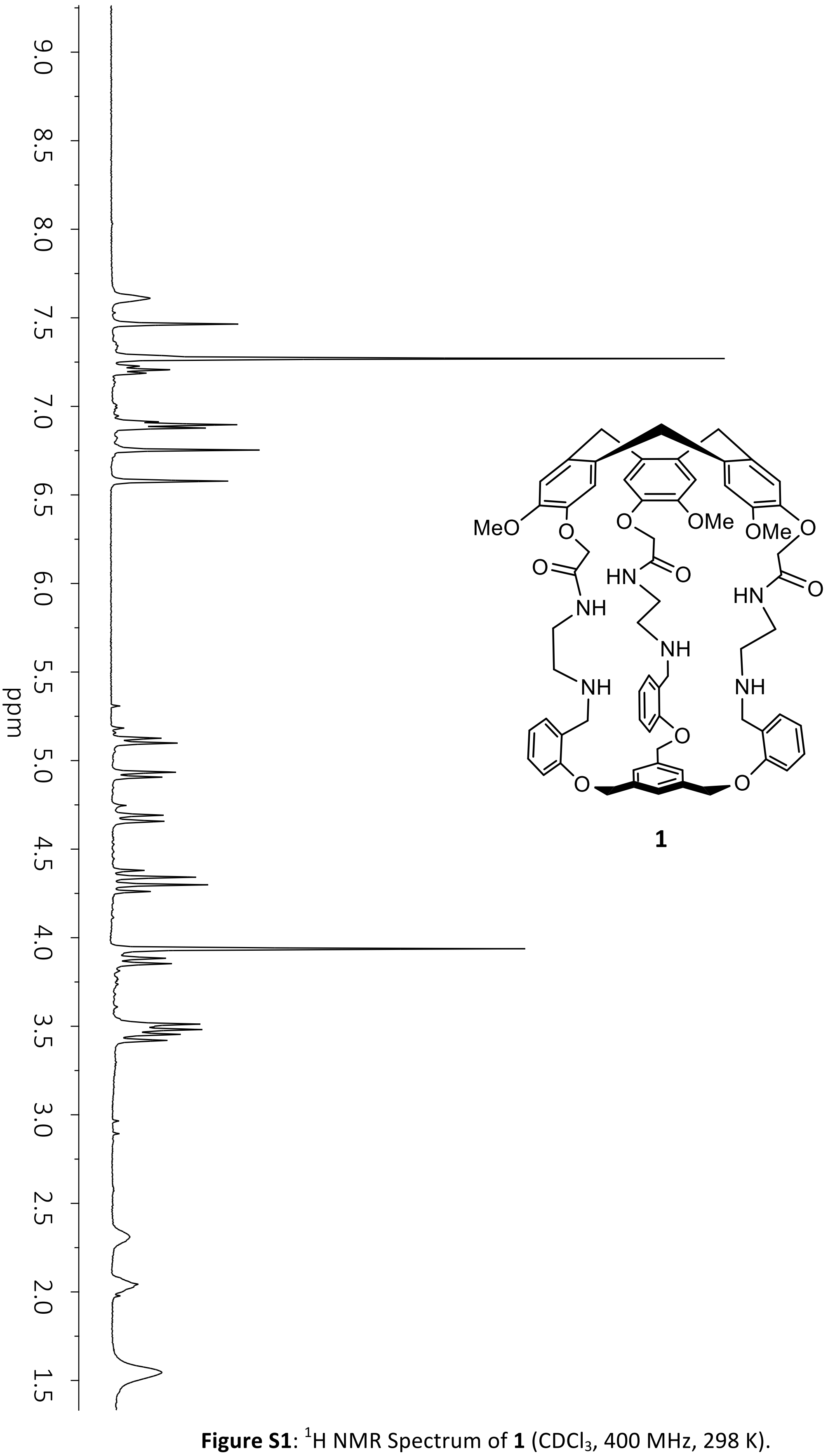

Figure S1: ^{1}H NMR Spectrum of **1** ($CDCl_3$, 400 MHz, 298 K).

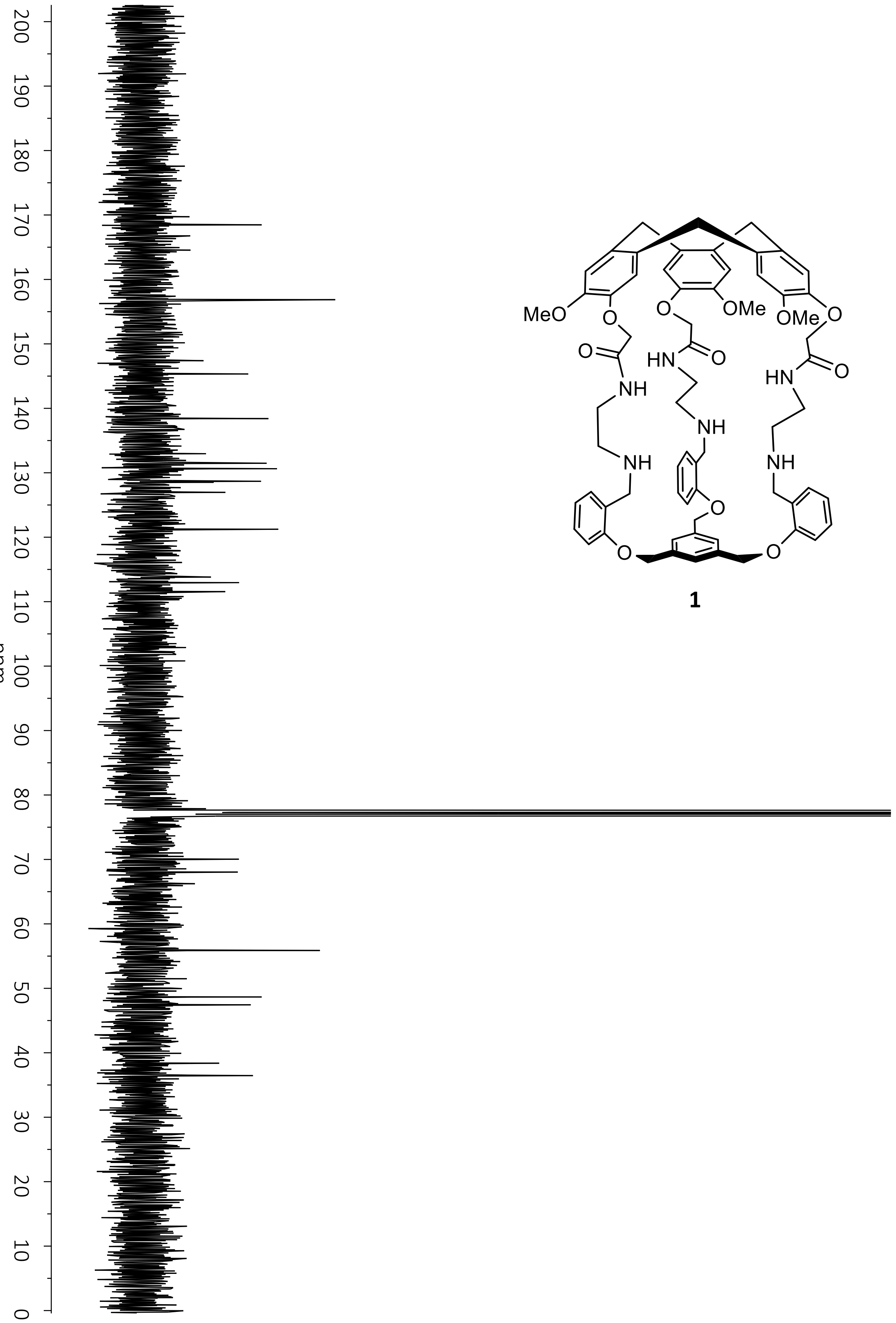

Figure S2: ^{13}C NMR Spectrum of **1** ($CDCl_3$, 100 MHz, 298 K).

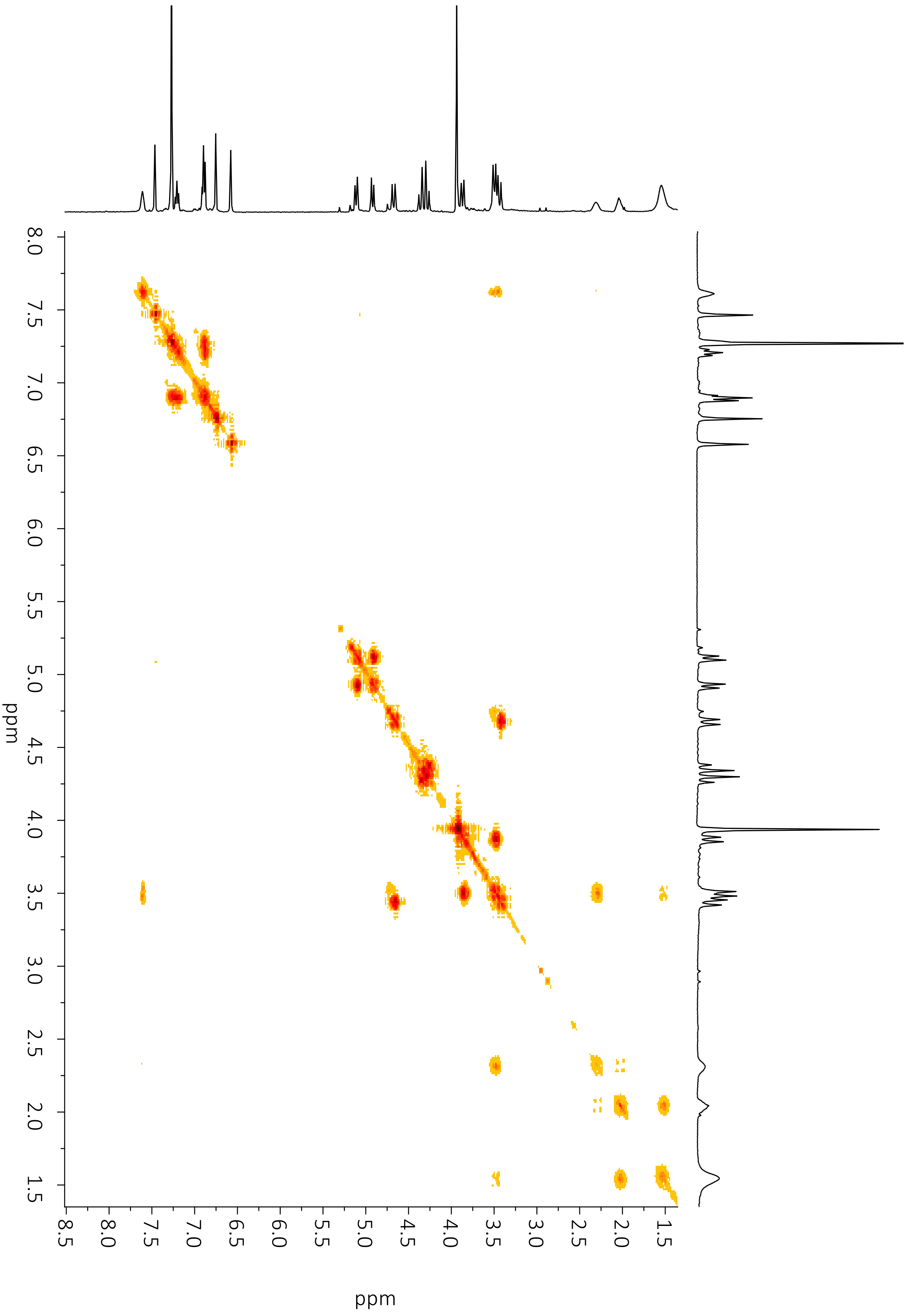

Figure S3: COSY Spectrum of **1** ($CDCl_3$, 400 MHz, 298 K).

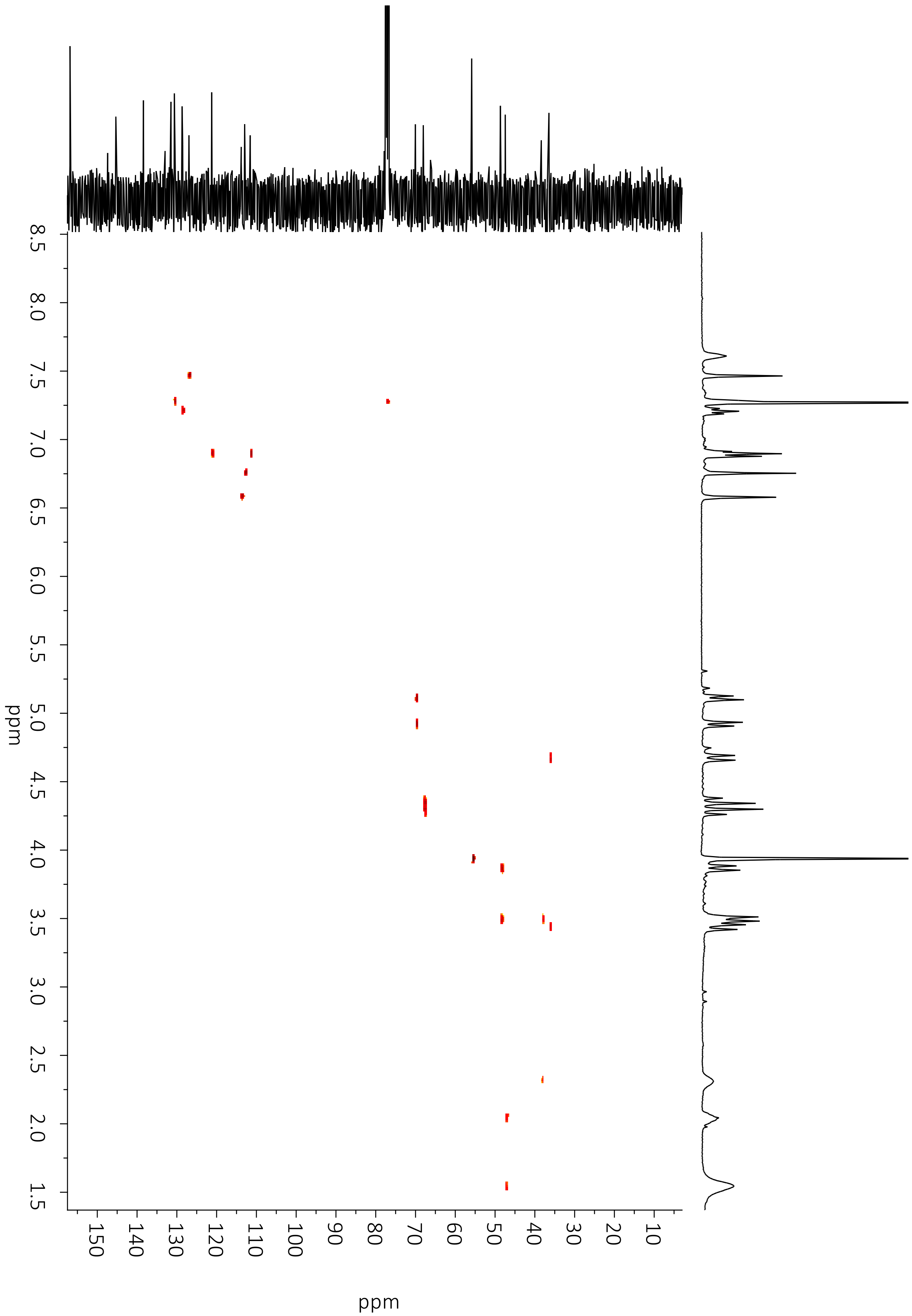

Figure S4: HSQC Spectrum of **1** ($CDCl_3$, 400 MHz, 298 K).

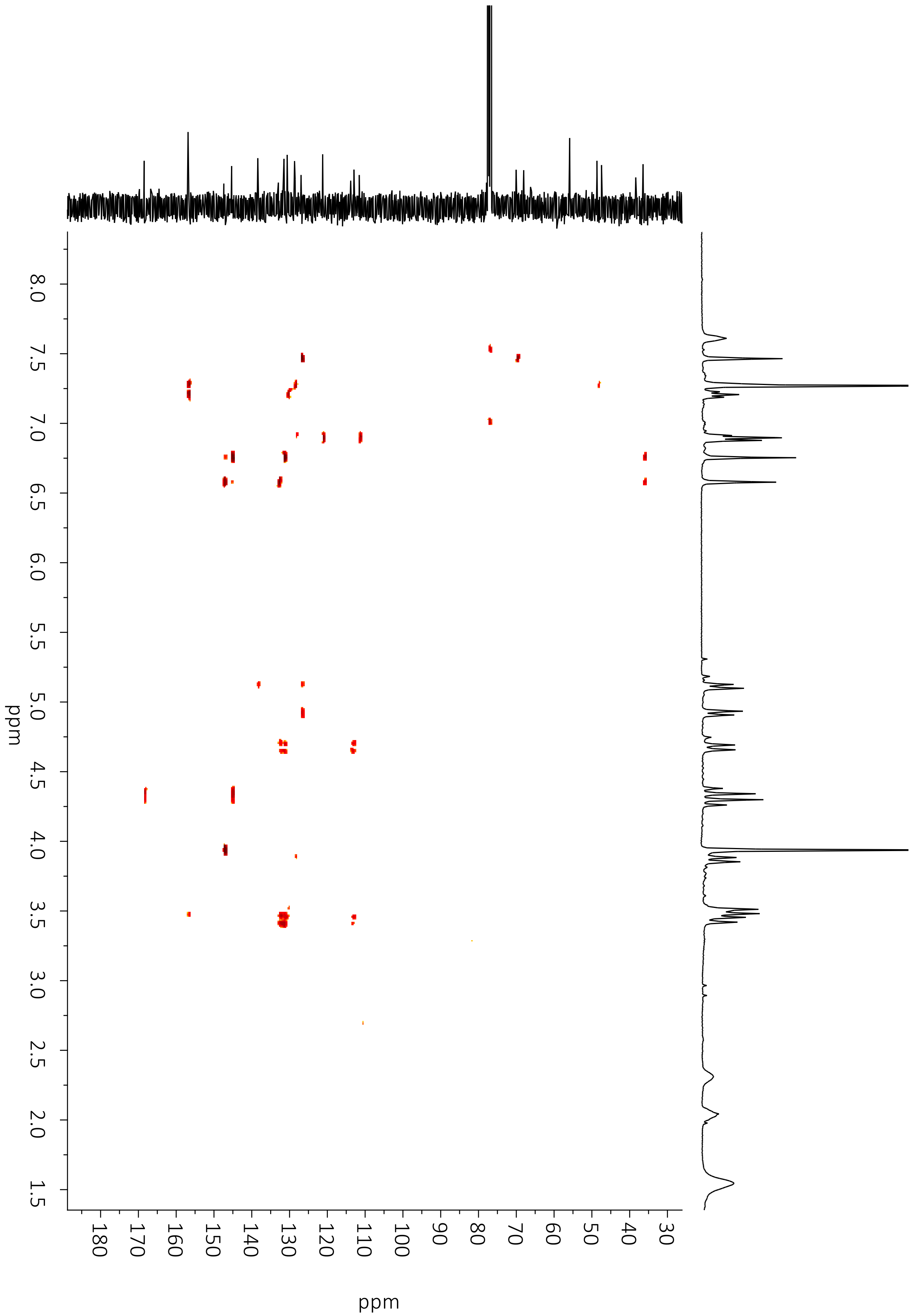

Figure S5: HMBC Spectrum of **1** ($CDCl_3$, 400 MHz, 298 K).

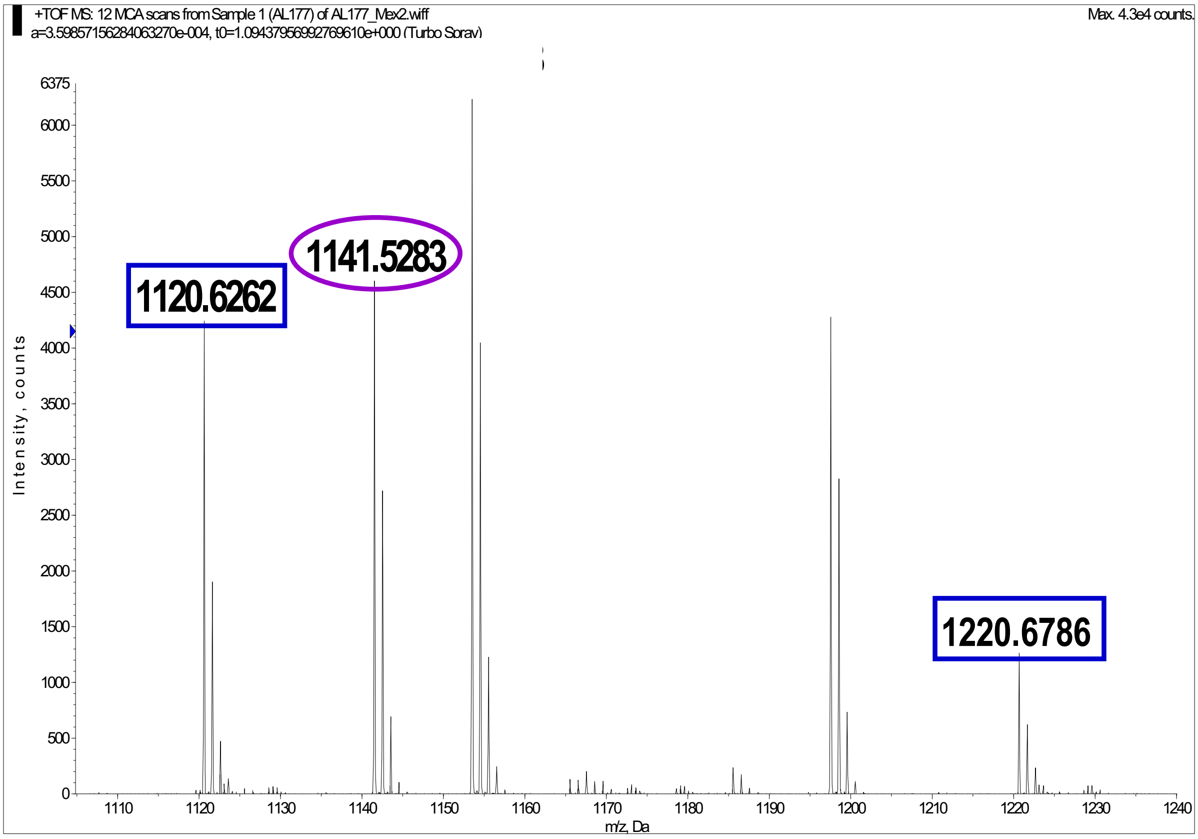

Figure S6: HRMS Spectrum of **1**+H^+. Expected peak is observed at 1141.5283 and internal standards NH_4^+ adducts with two oligomers of PMMA800 chosen for analysis at 1120.6262 and 1220.6786.

3. Crystallographic data for hemicryptophane 1

A suitable crystal of hemicryptophane **1** was selected and mounted on a Gemini kappa-geometry diffractometer (Rigaku OD, 2015) equipped with an Atlas CCD detector and using Cu radiation λ = 1.54180 Å). Intensities were collected at 150 K by means of the CrysalisPro software.[4] Reflection indexing, unit-cell parameters refinement, Lorentz-polarization correction, peak integration and background determination were carried out with the CrysalisPro software.[4] An analytical absorption correction was applied using the modeled faces of the crystal.[5] The resulting set of *hkl* was used for structure solution and refinement. The structures were solved by direct methods with SIR97,[6] and the least-square refinement on F^2 was achieved with the CRYSTALS software.[7] All non-hydrogen atoms were refined anisotropically. The hydrogen atoms were all located in a difference map, but those attached to carbon atoms were repositioned geometrically. The H atoms were initially refined with soft restraints on the bond lengths and angles to regularize their geometry (C–H in the range 0.93–0.98 Å, N–H in the range 0.86—0.89 Å) and U_{iso}(H) (in the range 1.2–1.5 times U_{eq} of the parent atom), after which the positions were refined with riding constraints. Inter molecular residual electronic density was located but could not be modelled. The contribution of the disordered solvent molecules was removed using the SQUEEZE algorithm.[8]

Table S1: Crystal Data and refinement parameters for **1**

formula	$C_{23}H_{25}Cl_3N_2O_4$
formula wt (g.mol^{-1})	499.02
crystal system	Trigonal
space group	*P* -3
a (Å)	16.763(5)
b (Å)	16.763(5)
c (Å)	15.231(5)
α (°)	90
β (°)	90
γ (°)	120
V (Å^3)	3706(2)
D_X	1.343
Z	6
F(000)	1560
μ (mm^{-1})	3.621
Temperature (K)	150
radiation λ (Å)	1.54180
$2\theta_{max}$ (deg)	62.132
Range of *hkl*	$-18 \leq h \leq 18$<br>$-19 \leq k \leq 19$<br>$-17 \leq l \leq 17$
no. of refl.	17835
no. of unique refl.	3889
no. of parameters	297
no. of restraints	8
R [I ≥ 2σ(*I*)]	0.1238
R_W[I ≥ 2σ(*I*)]	0.1623
$w = 1/(\sigma^2(Fo^2)+xP^2)$<br>where $P=(Fo^2+2Fc^2)/3$; x =	
goodness of fit	0.972
ρ_{min}/ρ_{max}	-1.20/1.08

Table S2: Atomic coordinates for **1**

	X	Y	Z
Cl1	0.2521(2)	0.2737(2)	0.46198(19)
C2	0.1759(8)	0.2465(8)	0.3712(8)
Cl3	0.0696(2)	0.1420(2)	0.3970(2)
Cl4	0.1569(2)	0.3329(2)	0.3421(2)
H21	0.2048	0.2337	0.3205
O5	0.6462(3)	0.5216(3)	-0.0390(3)
C6	0.7007(5)	0.5205(5)	-0.1108(5)
C7	0.6829(5)	0.4236(5)	-0.1174(5)
C8	0.5930(5)	0.3502(5)	-0.1192(5)
H81	0.5438	0.3605	-0.1201
H61	0.7650	0.5633	-0.0989
H62	0.6838	0.5391	-0.1636
C9	0.6706(5)	0.6035(5)	0.0005(5)
C10	0.6257(5)	0.5968(5)	0.0818(6)
C11	0.5570(5)	0.5019(4)	0.1165(5)
N12	0.6038(4)	0.4511(4)	0.1429(4)
C13	0.5482(5)	0.3683(5)	0.1911(5)
C14	0.4600(4)	0.2930(4)	0.1425(5)
N15	0.4144(4)	0.2111(4)	0.1950(4)
C16	0.3458(5)	0.1957(5)	0.2478(6)
O17	0.3094(3)	0.2442(3)	0.2522(4)
C18	0.3048(5)	0.1067(5)	0.3053(6)
O19	0.3627(3)	0.0684(3)	0.3155(4)
C20	0.4366(5)	0.1128(5)	0.3717(5)
C21	0.4917(5)	0.0700(5)	0.3788(5)
O22	0.4615(3)	-0.0101(3)	0.3335(3)
C23	0.5165(5)	-0.0521(5)	0.3403(6)
H231	0.4838	-0.1114	0.3122
H233	0.5273	-0.0604	0.4005
H232	0.5740	-0.0153	0.3107
C24	0.5692(5)	0.1135(5)	0.4313(5)
C25	0.5950(5)	0.1940(5)	0.4787(5)
C26	0.5374(5)	0.2333(5)	0.4756(5)
C27	0.5533(5)	0.3151(6)	0.5283(5)
H272	0.5915	0.3214	0.5788
H271	0.4933	0.3043	0.5466
C28	0.4595(5)	0.1919(5)	0.4194(5)
H281	0.4224	0.2183	0.4146
H241	0.6056	0.0857	0.4343
H182	0.2897	0.1209	0.3622
H181	0.2497	0.0610	0.2775
H151	0.437(3)	0.176(3)	0.197(4)

H142	0.4791	0.2778	0.0881
H141	0.4178	0.3143	0.1304
H132	0.5848	0.3416	0.2081
H131	0.5282	0.3856	0.2432
H121	0.623(3)	0.437(2)	0.0966(16)
H112	0.5298	0.5098	0.1686
H111	0.5086	0.4681	0.0734
C29	0.6494(5)	0.6759(5)	0.1306(6)
C30	0.7144(5)	0.7619(5)	0.0985(6)
C31	0.7545(5)	0.7693(5)	0.0177(7)
C32	0.7316(5)	0.6898(5)	-0.0334(6)
H321	0.7567	0.6943	-0.0876
H311	0.7980	0.8258	-0.0035
H301	0.7321	0.8142	0.1319
H291	0.6224	0.6720	0.1843
O5	0.4784(3)	0.1246(4)	-0.0390(3)
C6	0.4795(5)	0.1802(7)	-0.1108(5)
C7	0.5764(5)	0.2593(7)	-0.1174(5)
C8	0.6498(5)	0.2428(7)	-0.1192(5)
H81	0.6395	0.1833	-0.1201
H61	0.4367	0.2017	-0.0989
H62	0.4609	0.1447	-0.1636
C9	0.3965(5)	0.0671(7)	0.0005(5)
C10	0.4032(5)	0.0289(7)	0.0818(6)
C11	0.4981(4)	0.0551(6)	0.1165(5)
N12	0.5489(4)	0.1527(6)	0.1429(4)
C13	0.6317(5)	0.1799(7)	0.1911(5)
C14	0.7070(4)	0.1670(6)	0.1425(5)
N15	0.7889(4)	0.2033(6)	0.1950(4)
C16	0.8043(5)	0.1501(7)	0.2478(6)
O17	0.7558(3)	0.0652(4)	0.2522(4)
C18	0.8933(5)	0.1981(7)	0.3053(6)
O19	0.9316(3)	0.2943(4)	0.3155(4)
C20	0.8872(5)	0.3238(7)	0.3717(5)
C21	0.9300(5)	0.4217(7)	0.3788(5)
O22	1.0101(3)	0.4716(4)	0.3335(3)
C23	1.0521(5)	0.5686(7)	0.3403(6)
H231	1.1114	0.5952	0.3122
H233	1.0604	0.5877	0.4005
H232	1.0153	0.5893	0.3107
C24	0.8865(5)	0.4557(7)	0.4313(5)
C25	0.8060(5)	0.4010(7)	0.4787(5)
C26	0.7667(5)	0.3041(7)	0.4756(5)
C27	0.6849(6)	0.2382(8)	0.5283(5)
H272	0.6786	0.2701	0.5788
H271	0.6957	0.1890	0.5466

C28	0.8081(5)	0.2676(7)	0.4194(5)
H281	0.7817	0.2041	0.4146
H241	0.9143	0.5199	0.4343
H182	0.8791	0.1688	0.3622
H181	0.9390	0.1887	0.2775
H151	0.824(3)	0.261(4)	0.197(4)
H142	0.7222	0.2013	0.0881
H141	0.6857	0.1035	0.1304
H132	0.6584	0.2432	0.2081
H131	0.6144	0.1426	0.2432
H121	0.563(2)	0.186(4)	0.0966(16)
H112	0.4902	0.0200	0.1686
H111	0.5319	0.0405	0.0734
C29	0.3241(5)	-0.0265(7)	0.1306(6)
C30	0.2381(5)	-0.0475(7)	0.0985(6)
C31	0.2307(5)	-0.0148(7)	0.0177(7)
C32	0.3102(5)	0.0418(7)	-0.0334(6)
H321	0.3057	0.0624	-0.0876
H311	0.1742	-0.0278	-0.0035
H301	0.1858	-0.0821	0.1319
H291	0.3280	-0.0496	0.1843
O5	0.8754(4)	0.3538(3)	-0.0390(3)
C6	0.8198(7)	0.2993(5)	-0.1108(5)
C7	0.7407(7)	0.3171(5)	-0.1174(5)
C8	0.7572(7)	0.4070(5)	-0.1192(5)
H81	0.8167	0.4562	-0.1201
H61	0.7983	0.2350	-0.0989
H62	0.8553	0.3162	-0.1636
C9	0.9329(7)	0.3294(5)	0.0005(5)
C10	0.9711(7)	0.3743(5)	0.0818(6)
C11	0.9449(6)	0.4430(5)	0.1165(5)
N12	0.8473(6)	0.3962(4)	0.1429(4)
C13	0.8201(7)	0.4518(5)	0.1911(5)
C14	0.8330(6)	0.5400(4)	0.1425(5)
N15	0.7967(6)	0.5856(4)	0.1950(4)
C16	0.8499(7)	0.6542(5)	0.2478(6)
O17	0.9348(4)	0.6906(3)	0.2522(4)
C18	0.8019(7)	0.6952(5)	0.3053(6)
O19	0.7057(4)	0.6373(3)	0.3155(4)
C20	0.6762(7)	0.5634(5)	0.3717(5)
C21	0.5783(7)	0.5083(5)	0.3788(5)
O22	0.5284(4)	0.5385(3)	0.3335(3)
C23	0.4314(7)	0.4835(5)	0.3403(6)
H231	0.4048	0.5162	0.3122
H233	0.4123	0.4727	0.4005
H232	0.4107	0.4260	0.3107

C24	0.5443(7)	0.4308(5)	0.4313(5)
C25	0.5990(7)	0.4050(5)	0.4787(5)
C26	0.6959(7)	0.4626(5)	0.4756(5)
C27	0.7618(8)	0.4467(5)	0.5283(5)
H272	0.7299	0.4085	0.5788
H271	0.8110	0.5067	0.5466
C28	0.7324(7)	0.5405(5)	0.4194(5)
H281	0.7959	0.5776	0.4146
H241	0.4801	0.3944	0.4343
H182	0.8312	0.7103	0.3622
H181	0.8113	0.7503	0.2775
H151	0.739(4)	0.563(3)	0.197(4)
H142	0.7987	0.5209	0.0881
H141	0.8965	0.5822	0.1304
H132	0.7568	0.4152	0.2081
H131	0.8574	0.4718	0.2432
H121	0.814(4)	0.377(3)	0.0966(16)
H112	0.9800	0.4702	0.1686
H111	0.9595	0.4914	0.0734
C29	1.0265(7)	0.3506(5)	0.1306(6)
C30	1.0475(7)	0.2856(5)	0.0985(6)
C31	1.0148(7)	0.2455(5)	0.0177(7)
C32	0.9582(7)	0.2684(5)	-0.0334(6)
H321	0.9376	0.2433	-0.0876
H311	1.0278	0.2020	-0.0035
H301	1.0821	0.2679	0.1319
H291	1.0496	0.3776	0.1843

CCDC 1836454 contains the supplementary crystallographic data for **1**. These data can be obtained free of charge from The Cambridge Crystallographic Data Centre via www.ccdc.cam.ac.uk/data_request/cif.

References

[1] G. Vériot, J.-P. Dutasta, G. Matouzenko, A. Collet, *Tetrahedron* **1995**, *51*, 389–400.
[2] O. Perraud, V. Robert, H. Gornitzka, A. Martinez, J.-P. Dutasta, *Angew. Chem. Int. Ed.* **2012**, *51*, 504–508.
[3] S. Le Gac, X. Zeng, C. Girardot, I. Jabin, *J. Org. Chem.* **2006**, *71*, 9233–9236.
[4] CrysAlisPro, Agilent Technologies, Version 1.171.36.28 (release 20-01-2011 CrysAlis171 .NET) (compiled Feb 1 2013,16:14:44)
[5] Clark, R. C.; Reid, J. S. *Acta Cryst*. **1995**, *A51*, 887-897.
[6] Altomare, A.; Burla, M.C.; Camalli, M.; Cascarano, G. L.; Giacovazzo, C.; Guagliardi, A.; Grazia, A.; Moliterni, G.; Polidori, G.; Spagna, R. J. *App. Cryst.* **1999**, *32*, 115-119.
[7] Betteridge, P.W.; Carruthers, J. R.; Cooper, R. I.; Prout, K.; Watkin, D. J. J. *Appl. Cryst*. **2003**, *36,* 1487.
[8] P. v.d. Sluis; Spek, A. L. *Acta Cryst*. **1990**, *A46*, 194-201.
